# 685. Early Experience with a Simple Administration of a Novel Fecal Microbiome Replacement for Prevention of Recurrent *Clostridioides difficile*

**DOI:** 10.1093/ofid/ofad500.747

**Published:** 2023-11-27

**Authors:** Richard L Hengel, Sujatha Krishnan, Jonathan A Rosenberg, Timothy E Ritter, Kathy A Baker, Lucinda J Van Anglen, Amy Guo, Mielad Moosapanah, Min Yang, Kevin W Garey

**Affiliations:** Atlanta ID Group, Atlanta, Georgia; DFW Infectious Diseases, PLLC, Frisco, Texas; Medical Director of Research GI Alliance of Illinois, Highland Park, Illinois; GI Alliance, Southlake, Texas; Texas Christian University, Fort Worth, Texas; Healix Infusion Therapy, LLC, Sugar Land, Texas; Ferring Pharmaceuticals, parsippany, New Jersey; Ferring Pharmaceuticals, Inc., Parsippany, New Jersey; Analysis Group, Inc., Boston, Massachusetts; University of Houston, Houston, TX

## Abstract

**Background:**

Fecal Microbiota, live-jslm (RBL) is a rectally administered, pre-packaged, live biotherapeutic approved in November 2022 for the prevention of recurrence of *Clostridioides difficile* infection (rCDI) in adults. As the first FDA-approved microbiota product, this new agent may pose challenges in administration, particularly with Infectious Disease (ID) or other specialties not typically providing rectally administered therapies. As part of an ongoing real-world study of RBL, the purpose of this study is to develop and report a simple protocol for the administration of RBL in clinical practice.

**Methods:**

We reviewed electronic health records, administration records and internal databases for patients receiving RBL since February 2023. Records were reviewed for patient demographics, setting of care, time from order to treatment, logistics from order to administration and payor details.

**Results:**

Six rCDI patients (73±14 years; female: 67%) have received RBL; 1 treated in an ID practice and the other 5 in GI practices. Following insurance approval and scheduling of the patient, RBL was ordered from the distributor and shipped to the practices in a 150mL pre-packaged frozen suspension. RBL was thawed for 24 hours and administered within 5 days. Average time from order to treatment was 18±5 days. Administration of RBL was performed by registered nurses or licensed vocational nurses. Reported RBL administration time was 5 minutes, with an additional 15 minutes for observation. Medicare (MCR) was the most common payor (71%) with traditional MCR (n=3), MCR Advantage (n=2), and one commercially insured. There have been no payor denials. Criteria for approval differed slightly between payors, but all required at least 1 prior episode of CDI. All plans required prior authorization except traditional MCR. Reimbursement was adequate for both the medication and instillation protocols.

**Conclusion:**

Early experience with RBL administration found more treatment by GI than ID physician practices. Authorizations were granted as expected with the administration and visit surprisingly brief and simple. RBL holds promise as a feasible office-based therapy for prevention of rCDI.

**Disclosures:**

**Jonathan A. Rosenberg, MD**, Aimmune: Advisor/Consultant|Ferring Pharmaceuticals: Advisor/Consultant **Timothy E. Ritter, MD**, Abbvie: Advisor/Consultant|Ardelyx: Advisor/Consultant|Arena: Advisor/Consultant|Boehringer Ingelheim: Advisor/Consultant|Bristol Myers Squibb/Celgene: Advisor/Consultant|Eli Lilly: Advisor/Consultant|Ferring: Advisor/Consultant|Ferring: Data Adjudication Committee|Genetech/Roche: Advisor/Consultant|Gilead: Advisor/Consultant|Intercept: Advisor/Consultant|Iterative Scopes: Expert Testimony|Iterative Scopes: Ownership Interest|Janssen: Advisor/Consultant|Nestle/Seres: Advisor/Consultant|Pfizer: Advisor/Consultant|Prometheus: Advisor/Consultant|Rebiotix: Data Adjudication Committee|Sanofi: Advisor/Consultant|Takeda Pharmaceuticals: Advisor/Consultant **Lucinda J. Van Anglen, PharmD**, ADMA Biologics, Inc.: Grant/Research Support|Ferring Pharmaceuticals: Grant/Research Support|Novartis: Grant/Research Support|Octapharma: Grant/Research Support|Paratek Pharmaceuticals: Grant/Research Support **Amy Guo, PhD**, Ferring Pharmaceuticals: Employee **Mielad Moosapanah, PharmD**, Ferring Pharmaceuticals: Employee **Min Yang, MD, PhD**, Analysis Group, Inc.: I am an employee of Analysis Group, Inc., which has received consulting fees from Ferring for the conduct of this study. **Kevin W. Garey, PharmD, MS**, Acurx: Grant/Research Support|Ferring: Advisor/Consultant|Paratek: Grant/Research Support

